# Effects of the Temperature–Time Regime of Curing of Composite Patch on Repair Process Efficiency

**DOI:** 10.3390/polym13244342

**Published:** 2021-12-11

**Authors:** Andrii Kondratiev, Václav Píštěk, Lina Smovziuk, Maryna Shevtsova, Anna Fomina, Pavel Kučera, Aleš Prokop

**Affiliations:** 1 Department of Building Technology and Construction Materials, O.M. Beketov National University of Urban Economy in Kharkiv, Marshal Bazhanov Str. 17, 61002 Kharkiv, Ukraine; andrii.kondratiev@kname.edu.ua; 2 Institute of Automotive Engineering, Brno University of Technology, Technická 2896/2, 616 69 Brno, Czech Republic; pistek.v@fme.vutbr.cz (V.P.); kucera@fme.vutbr.cz (P.K.); 3 Department of International Projects and Programs, National Aerospace University “Kharkiv Aviation Institute”, Chkalova Str. 17, 61070 Kharkiv, Ukraine; l.smovziuk@khai.edu; 4 Department of Composite Structures and Aviation Materials, National Aerospace University “Kharkiv Aviation Institute”, Chkalova Str. 17, 61070 Kharkiv, Ukraine; m.shevtsova@khai.edu; 5 Department of Railway, Automobile Transport and Handling Machines, Institute of Transport and Logistics, Volodymyr Dahl East Ukrainian National University, Central Avenue 59a, 93400 Sewerodonetsk, Ukraine; anyta220885@gmail.com

**Keywords:** temperature–time regime, binder shrinkage, gelation

## Abstract

Repair procedures with the use of composite patches are considered to be the most effective among the current technologies of repair of the structures of various applications. In the process of moulding-on of a patch made of polymeric composite material by means of curing, technological stresses arise in the patch. Determination of residual technological stresses is a priority task for the modelling of the repair process. Reduction of residual stresses can be achieved by optimization of the mode of repair patch curing. For meeting this objective, the method for determination of technological stresses, which arise in the structure under repair in the process of curing of a composite patch, has been developed. The method takes into account the shrinkage, change in physico-mechanical characteristics, rheological processes occurring in the binder during moulding process, and determination of stresses in the structure under repair at any time. Therefore, premature failure of the repair joint at the stage of repair can be avoided. It is shown that the method adequately describes the level of deformations and stresses in the structure being repaired at the stage of heating and holding of the composite patch. Increase in the moulding temperature leads to a reduction in residual stresses in the structure under repair. However, current stresses at the stages of heating and temperature holding are increased significantly. Reliability of assumptions and developed method is confirmed by the comparison with the experimental data. The obtained experimental graph of total deformation of the composite patch allowed us to clearly determine the moment of residual stress occurrence in the structure under repair. This moment matches quite exactly (with the discrepancy not exceeding 5 min) the gel point determined analytically based on dependence of the degree of curing on the moulding mode. Consequently, the research together with the results previously obtained allows making an integrated choice of geometric parameters of the repair composite patch and temperature–time regime of its curing in order to ensure the specified level of strength and stiffness of the structure under repair.

## 1. Introduction

In the process of operation of various structures, defects may arise in a number of situations, and development of such defects leads to damage that prevents further use of the structure [1,2]. Effective repair of any detected defects, including the repair in the field conditions, allows for the protection of the damaged elements [3,4] and for the avoidance of lengthy and costly capital repairs [5,6].

According to research [7,8], installation of repair patches is considered an effective method for the repair of defects such as dents, failures of bearing skins and delaminations in the metal and composite panel structures. High physico-mechanical characteristics of the modern polymeric composite materials (PCM) [9,10,11] determine successful use of the composite patches for the repair of both polymeric and metal structures [12,13]. The composite patch can also have potential applications in constructing electromagnetic nanocomposites or intelligent materials, as there is carbon materials in the composite [14,15]. However, stresses occurring in the process of curing the repair patch further affect the bearing capacity of repaired product [16]. Presence of unrecorded initial stresses in the repaired structure causes the appearance of defects such as delaminations, buckling and microcracks in the binder [17,18], which in turn has a significant effect on the static and fatigue strength of the product made with such structures [19,20].

Reduction of residual stresses can be achieved by optimization of the mode of repair patch curing. Technological stresses are caused by shrinkage of the polymeric binder, as well as the difference in the linear thermal expansion coefficients of materials to be joined [21]. Curing of thermo-reactive polymeric binder as the most commonly used binder in the repair patches of various applications [22] is, in fact, the combination of a number of physico-chemical processes. Their mutual influence determines the behaviour of the binder and the nature of changes in its main characteristics. Assessment of the influence of parameters of the curing mode on the stress–strain behaviour of the structure under repair and choice of their rational values can be performed only with a set of interrelated mathematical models to determine the current values of viscosity, shrinkage, degree of curing and mechanical characteristics of the PCM at any time. A significant part of papers such as [23,24] is focused on the study of kinetics of thermo-reactive binder curing and analytical modelling of the change in the curing degree depending on the temperature–time parameters of the process. This focus is driven by the fact that achievement of high curing rates is a prerequisite for the manufacturing of high-strength composite structures [25]. The variety of phenomenological models of curing kinetics found in the literature is explained by significant differences in the behaviour of various binders [26,27].

The simplified model of the binder in the process of curing, taking into account stress relaxation, is proposed in [28]. This model provides the balance of the modelling accuracy and efficiency, which is extremely necessary in practice.

Modelling and analysis of the thermo-reactive binder curing taking into account gradual changes in the thermal and mechanical properties is presented in [29]. It is shown that temporal and spatial changes in the physical properties of epoxy resin in the course of curing affect the elastic modulus of the cured PCM specimens.

The paper [30] presents a viscoelastic model of epoxy resin, depending on temperature and degree of curing. The results were obtained based on the hypothesis of time and temperature overlap. The effect of the degree of curing was taken into account through glass transition temperature, dependent on the curing, used as a reference temperature for the shear coefficients.

The pattern for modelling of residual stresses and technological deformations in the PCM is proposed in [31]. Experimental comparison of the modelling results for all mechanical characteristics at all stages of the phase transformation of the material is given. Unfortunately, the equations obtained for modelling of all key processes associated with the temperature cycle of hardening of the thermoplastic matrix are presented together with the corresponding material constants using the example of polyetheretherketone (PEEK) only.

The method for predicting the distribution of residual stresses in PCM caused by the technological process in the course of binder curing is proposed in [32]. Distributions of the temperature and degree of curing were obtained. The elastic modulus of the binder was determined using a model of instantaneous linear elasticity at curing.

The analytical model for recording of thermoviscoelastic effects on the residual stresses of composite parts during the curing process is developed in [33]. Viscoelastic effects on the residual stresses and elasticity due to tangential and radial thermochemical expansion are considered separately in order to help understand their individual influence.

The papers [34,35] deal with the change in distribution of the field of residual stresses in the specimen of material AS4/8552-1 with the lay-up of (0°/90°) in the process of curing. To simulate the curing, the coupled thermal and strength problem is solved under plane deformation conditions.

A common disadvantage of the papers considered is that they focus only on the method for stress determination, without paying due attention to the curing process itself. As a result, they do not take into account the change in the PhMC of the composite material during the curing process, and consider the residual stresses caused by the temperature component, only without taking into account the shrinkage. In many papers, only the cooling stage is considered, i.e., stresses arising during the heating process are unreasonably neglected. An exception is [36], where the stress–strain behaviour is determined, taking into account the previously developed kinetic model of the binder performance in the curing process, as well as [34,35], providing the model for behaviour of viscoelastic material to describe the PCM operation in the manufacturing process, including the processes of formation, polymerization, and development of residual deformations and stresses.

The results of a number of experimental studies are of great importance for understanding the mechanisms of occurrence of temperature stresses in the process of curing of the repair patch and possible ways to reduce them [37,38]. Study of the temperature deformations occurring in the structure under various temperature and time conditions of polymerization are dealt with in [39,40]. However, the experimental measurement of residual stresses at curing is often an expensive and difficult process [41]. Therefore, results of experimental studies in a number of works are supplemented with the data obtained using the finite element modelling [42,43]. It allowed the authors to give some recommendations regarding the choice of optimal parameters for the repair process [44]. The paper [45] proposes a multiscale model for prediction of residual stresses of composites in the process of curing. However, the main disadvantage of the numerical approach is that the finite element model is created for a strictly defined structure and cannot be arbitrarily extended to similar elements and manufacturing processes [46].

Thus, it can be said that, even as the problem of determining the residual technological stresses in panels repaired by a composite patch is discussed in modern literature from various positions, the studies considered do not take into account a number of significant factors.

**Figure 1:**
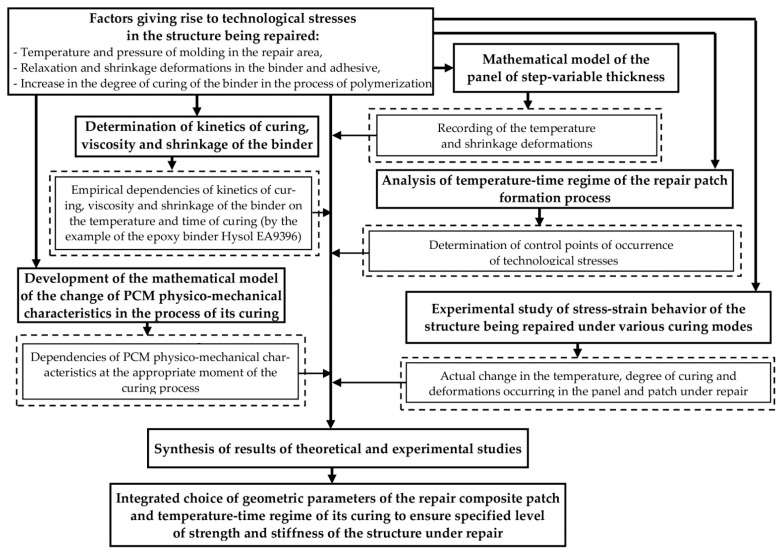
The research flow chart.

The objective of the work is to develop a method for determining the technological stresses arising in the structure under repair in the process of curing of the composite patch, which would take into account the shrinkage, changes in physico-mechanical characteristics and rheological processes occurring in the binder during the moulding process. Figure 1 shows the research flow chart.

## 2. Materials and Methods

To determine the stress–strain behaviour of the structure in the process of curing of the composite patch, advanced mathematical model of a panel of step-variable thickness was used [47]. The mathematical model for the change in the PCM physical and mechanical characteristics in the process of curing of the composite patch was developed for taking into account the technological stresses arising in the process of the composite patch moulding-on. In the framework of this model, for the determination of kinetics of curing, viscosity and shrinkage of the binder, empirical dependences of these parameters on the temperature and time of curing obtained in [48,49] on the results of isothermal and dynamic calorimetric tests for the epoxy binder Hysol EA9396 (Henkel, Bay Point, CA, USA) were used. In the process of construction of the mathematical model, taking into account a sharp change in the binder viscosity during transition from the liquid to the elastic state, it was assumed that technological stresses would arise in the structure after gelation of the binder only. The linear dependence of the elastic modulus on the degree of curing was taken. Additionally, it was assumed that Poisson’s ratio and coefficient of linear thermal expansion of the binder were not dependent on the degree of curing, with the linear thermal expansion coefficient being the known function of temperature. Dependences of the current elastic characteristics of the monolayer at a particular moment of curing on the characteristics of the fibre and matrix were determined from the known dependences of the reinforced media mechanics [50,51]. Woven reinforcing material was considered as a package, consisting of two identical monolayers with laying angles of [0°, 90°]. Technological stresses in the structure under repair were determined as a sum of stresses arising in separate time intervals, within which the temperature and shrinkage stresses were caused by corresponding changes in temperature and shrinkage; the elastic modulus and the coefficient of linear thermal expansion of the binder were constant.

The number of areas of division was determined by the required computational accuracy. Reliability of the constructed mathematical model is confirmed by comparison with the results of experimental studies of deformations arising in the aluminium panel and carbon fibre composite patch in the moulding-on process. To do this, a carbon fibre composite patch (EA9396/Carbon 3K-70-P, Henkel, Bay Point, CA, USA) with a lay-up pattern of (0°; 90°) was laid on prefabricated specimen of the aluminium alloy 2024T3 (TW Metals Corporate Headquarters, Exton, PA, USA) by wet method. Thickness of the patch after curing was 2.2 ± 0.07 mm. Deformations arising in the tested structure in the process of curing of the composite patch were measured using KF5P1-20-400 (VEDA Group of Companies, Kyiv, Ukraine) strain gauges. To implement the selected curing modes of the repair patch, the thermal oven providing the uniform heating over the entire area of the specimen was used. For more accurate temperature control, an additional thermocouple was installed directly on the test specimen. Due to small thickness of the specimen and two-way heating provided in the furnace, uneven temperature distribution over the thickness was not taken into account. To prevent the impact of the internal temperature deformations of the strain gauge on the results of the experiment, strain gauges were connected to the measuring system taking into account the thermal compensation

## 3. Theoretical Background

As stated above, it is not possible to construct the general curing model suitable for any thermo-reactive binder [23,24]. This is due to significant difference in the behaviour of various types of polymers [22].

**Figure 2:**
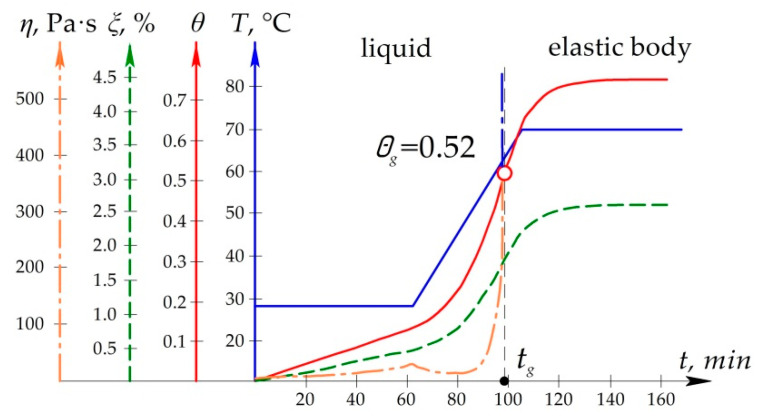
Change in viscosity (*η*), shrinkage (*ξ*) and degree of curing (*θ*) of the binder EA 9396 at the curing mode recommended by the manufacturer [48,49].

Epoxy binder Hysol EA9396 (Henkel), widely used for repairs in the foreign aviation industry, was chosen for the research. To determine the kinetics of curing, viscosity and shrinkage of the binder EA 9396, empirical dependences constructed in [48,49] under the international project SENARIO (AST5-CT-2006-030982) with the financial support of the European Commission were used, based on the results of isothermal and dynamic calorimetric tests and experimental study of shrinkage. Figure 2 shows the graphs of changes in the abovementioned characteristics at the curing mode recommended by the binder manufacturer.

With the use of these models and based on the fact that a sharp change in the binder viscosity corresponds to its transition from the liquid to elastic state [25], calculations showed that, at the time of gelation, the degree of the binder curing was equal to 52%.

With the increase in the degree of the binder curing, the change in its elastic characteristics occurs [26,27,29].

Assuming the linear dependence of the elastic modulus on the degree of curing and taking into account the sharp change in the elastic modulus during passing through the gel point experimentally proven in [50], we use the function below for setting of the binder elastic modulus

(1) $$E_{b}\left(t\right)=\{\begin{array}{c} 0,\;\;\;\;\;\;\;\;\;\;\;\;\;\;\;t<t_{g}\\ E_{b}^{\theta =100\%}\theta ,\;t\geq t_{g} \end{array},\;$$

where *θ*—current value of the degree of curing; *t_g_*—gelation time; $E_{b}^{\theta =100\%}$—binder elastic modulus at 100% degree of curing.

Assuming that after gelation the Poisson’s ratio and the binder linear thermal expansion coefficient change insignificantly, we consider them independent of the degree of curing. However, we take into account a significant change in the binder linear thermal expansion coefficient on the temperature *T*. Dependence of the elastic characteristics of initial PCM components (fibre and matrix) on the temperature will be neglected because of a slight change in these properties at low curing temperatures [22]. Dependences of the current elastic characteristics of the monolayer at a particular moment of curing on the characteristics of the fibre and matrix were determined using the known dependencies [51,52]:

(2) $$\begin{array}{c} E_{1}\left(t\right)=E_{f}\Theta +E_{b}\left(t\right)\left(1-\Theta \right),\;E_{2}\left(t\right)=\frac{E_{f}E_{b}\left(t\right)}{E_{b}\left(t\right)\Theta +E_{f}\left(1-\Theta \right)},\\ G_{12}\left(t\right)=\frac{G_{f}G_{b}\left(t\right)}{G_{b}\left(t\right)\Theta +G_{f}\left(1-\Theta \right)},\;\mu_{12}\left(T\right)=\mu_{f}\Theta +\mu_{b}\left(1-\Theta \right),\\ \alpha_{1}\left(t\right)=\frac{E_{f}\alpha_{f}\Theta +E_{b}\left(t\right)\alpha_{b}\left(T\right)\left(1-\Theta \right)}{E_{f}\Theta +E_{b}\left(t\right)\left(1-\Theta \right)},\\ \alpha_{2}\left(t\right)=\frac{\left(\alpha_{f}\Theta +\alpha_{b}\left(T\right)\left(1-\Theta \right)\right)\left(E_{f}\Theta +E_{b}\left(t\right)\left(1-\Theta \right)\right)}{E_{f}\Theta +E_{b}\left(t\right)\left(1-\Theta \right)}+\\ \frac{\Theta \left(1-\Theta \right)\left(\alpha_{b}\left(T\right)-\alpha_{f}\right)\left(E_{f}\mu_{b}-E_{b}\left(t\right)\mu_{f}\right)}{E_{f}\Theta +E_{b}\left(t\right)\left(1-\Theta \right)},\\ \xi_{1}\left(t\right)=\frac{E_{b}\xi_{b}\left(1-\Theta \right)}{E_{f}\Theta +E_{b}\left(t\right)\left(1-\Theta \right)},\\ \xi_{2}\left(t\right)=\frac{E_{f}\Theta +E_{b}\left(t\right)\left(1-\Theta \right)+\Theta \left(E_{f}\mu_{b}-E_{b}\left(t\right)\mu_{f}\right)}{E_{f}\Theta +E_{b}\left(t\right)\left(1-\Theta \right)}\left(1-\Theta \right)\xi_{b}, \end{array}\}$$

where *E_f_*, *E_b_*, *G_f_*, *G_b_*, *μ_f_*, *μ_b_*, *ξ_f_*, *ξ_b_*—elastic modulus, shear modulus, Poisson’s ratios, fibre and binder shrinkage, accordingly; Θ—relative volumetric content of fibres.

Woven reinforcing material is considered as a package consisting of two identical monolayers with laying angles of [0°, 90°], for which [34,46]:

(3) $$\begin{array}{c} E_{x}=E_{y}=\frac{{\bar{E}}_{1}}{2}+\frac{{\bar{E}}_{2}}{2}-2\frac{{\bar{E}}_{1}^{2}\mu_{21}^{2}}{{\bar{E}}_{1}+{\bar{E}}_{2}};G_{xy}=G_{12},\\ \alpha_{x}=\alpha_{y}=\frac{\left(\frac{\alpha_{1}{\bar{E}}_{1}}{2}+\frac{\alpha_{2}{\bar{E}}_{2}}{2}+\frac{\alpha_{1}+\alpha_{2}}{2}{\bar{E}}_{1}\mu_{21}\right)\left(\frac{{\bar{E}}_{1}}{2}+\frac{{\bar{E}}_{2}}{2}-{\bar{E}}_{1}\mu_{21}\right)}{{\left(\frac{{\bar{E}}_{1}}{2}+\frac{{\bar{E}}_{2}}{2}\right)}^{2}-{\bar{E}}_{1}^{2}\mu_{21}^{2}},\\ \xi_{x}=\xi_{y}=\frac{\left(\frac{\xi_{1}{\bar{E}}_{1}}{2}+\frac{\xi_{2}{\bar{E}}_{2}}{2}+\frac{\xi_{1}+\xi_{2}}{2}{\bar{E}}_{1}\mu_{21}\right)\left(\frac{{\bar{E}}_{1}}{2}+\frac{{\bar{E}}_{2}}{2}-{\bar{E}}_{1}\mu_{21}\right)}{{\left(\frac{{\bar{E}}_{1}}{2}+\frac{{\bar{E}}_{2}}{2}\right)}^{2}-{\bar{E}}_{1}^{2}\mu_{21}^{2}}, \end{array}\}$$

where ${\bar{E}}_{1}=\frac{E_{1}}{1-\mu_{12}\mu_{21}}$, ${\bar{E}}_{2}=\frac{E_{2}}{1-\mu_{12}\mu_{21}}$.

**Figure 3:**
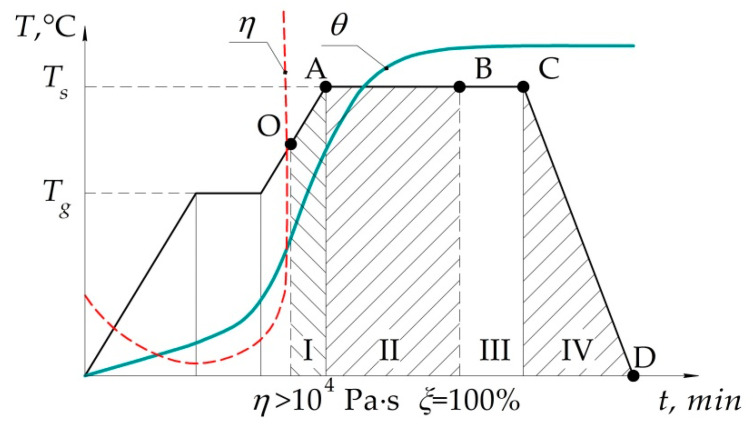
Typical temperature–time dependence of the polymeric binder curing mode.

In order to determine the stress–strain behaviour of the structure under repair, we apply the analytical model of a panel of step-variable thickness [19]. Now we shall analyse the external and internal force factors leading to the appearance of stresses in the structure under repair, using the example of typical temperature–time dependence of the polymeric binder curing mode (Figure 3) [19,22].

Taking into account that technological stresses occur in the structure only after gelation (point O) [25,29,31], the typical curing process can be roughly divided into four stages, as shown in Figure 3.

At the first stage, occurrence and growth of stresses is determined by heating and available shrinkage deformations. When metal structures are repaired using the composite patch, thermal and shrinkage stresses can have the same sign at the definite ratio of the linear thermal expansion coefficient and stiffnesses [19]. In this case, temperature stresses supplemented with shrinkage ones may lead to occurrence of defects already in the heating process, since physico-mechanical characteristics of the patch material at the first stage are considerably lower than the final values. It determines the need to control the stresses at point A.

During the isothermal holding (AC), stresses result from the continuing shrinkage (II) and relaxation processes (III) [28,32,33].

At the fourth stage, the occurring stresses are determined by cooling of the structure being repaired and the ongoing relaxation processes. The final technological stresses at point D summarize the stresses at all previous stages and are subject to control [22].

Under certain conditions, at each stage the effective voltages can exceed the current permissible values. It determines the need to control both the residual stresses (point D), and the current stresses at design points A, B and C [47].

In order to determine the stress–strain behaviour of the structure with the characteristics changing in the curing process, arising stresses are determined as a sum of stresses on the certain areas, within which [19]:

- Thermal and shrinkage stresses are caused by corresponding increase in the temperature and shrinkage
  (4) $$\Delta T_{i}=T_{i+1}-T_{i};\;\Delta \xi_{i}=\xi_{i+1}-\xi_{i};$$
- Elastic modulus of the binder is taken to be constant and equal to
  (5) $$E_{b}^{i}=\frac{1}{t_{i+1}-t_{i}}\int_{t_{i}}^{t_{i+1}}E\left(t\right)dt;$$
- The binder linear thermal expansion coefficient is taken to be constant and equal to
  (6) $$\alpha_{b}^{i}=\frac{1}{T_{i+1}-T_{i}}\int_{T_{i}}^{T_{i+1}}\alpha \left(T\right)dT.$$

The number of areas of division is determined by the required computational accuracy.

The basis for optimization of the structure repair using PCM is the analysis of the influence of individual parameters of the repair patch curing process. They can be roughly divided into two groups:

- Curing process parameters;
- Repair process parameters.

Among the parameters of the moulding mode, significant effect on the stress–strain behaviour is exerted, first of all, by curing temperature ($T_{c}$), heating rate ($V_{1}$) and cooling rate ($V_{2}$) of the structure under repair. Holding time at $T_{c}$ determines the degree of curing of the repair patch and affects not so much the stress–strain behaviour, but physico-mechanical characteristics of the PCM.

The second group of parameters should include the conditions for the structure fixation during the repair process, method for implementing the temperature–time regime, and presence of additional external loads.

**Figure 4:**
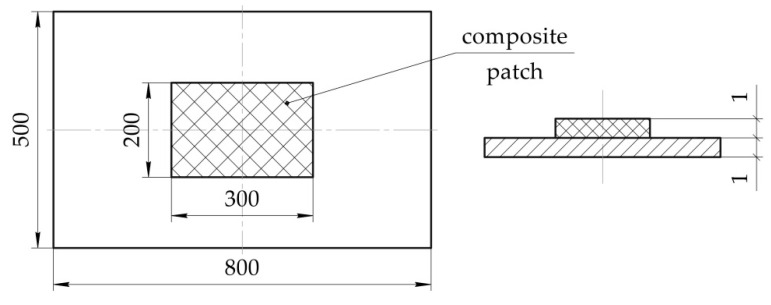
Geometric parameters of the investigated structure under repair.

Let’s investigate the stress–strain behaviour of the flat rectangular panel (Figure 4) made of the aluminium alloy 2024T3, of the parameters below:

- Curing temperature $T_{c}$;
- Heating rate $V_{1}$;
- Conditions for the structure fixation in the repair process;
- Heating method.

Repair of the structure under study is carried out by installing a rectangular carbon fibre composite plate. Physico-mechanical characteristics of the initial PCM components are given in Table 1 [49].

For the modes presented in Table 2, stress–strain behaviour occurring during repair of the analysed structure in a free state has been determined. In this case, the boundary conditions corresponded to the hinge mount of the panel at the central point, ensuring its unrestricted deformation in the moulding process. The uniform temperature setting over the entire structure area modelled the patch curing in the oven. Non-uniformity of temperature over the thickness was not taken into account because of the small thickness of parts and the presence of a two-way heat supply.

**Figure 5:**
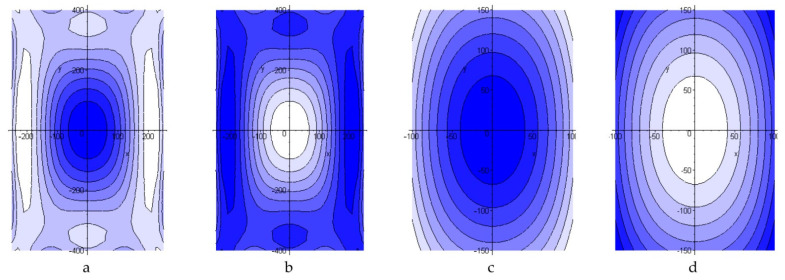
Distribution of normal stresses *σ_x_* in the aluminium panel (**a**,**b**) and repair patch (**c**,**d**) on completion of the temperature holding at *T_c_* and cooling.

**Figure 6:**
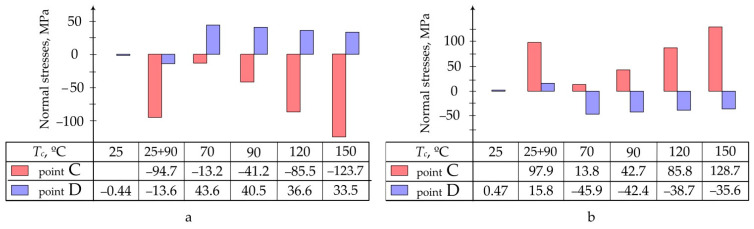
Maximum normal stresses occurring in the aluminium panel (**a**) and composite patch (**b**) at different curing temperatures.

**Figure 7:**
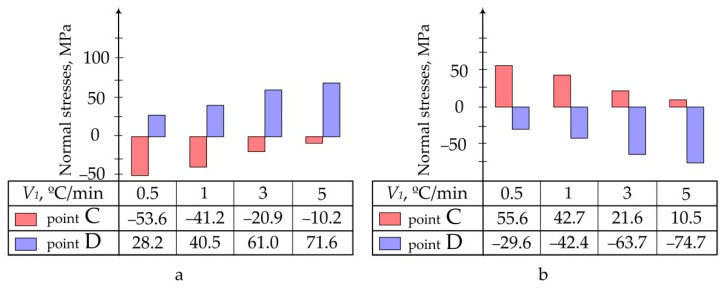
Maximum normal stresses occurring in the aluminium panel (**a**) and composite patch (**b**) at different heating rates (*V*_1_).

Data obtained as a result of calculations are presented in the form of:

- Pictures of stress distribution in the panel and patch at the stage of temperature holding and after cooling of the structure (Figure 5);
- Diagrams of maximum stresses in the structure during moulding at different temperatures (Figure 6);
- Diagrams of maximum stresses in the structure during moulding at different heating rates (Figure 7).

**Figure 8:**
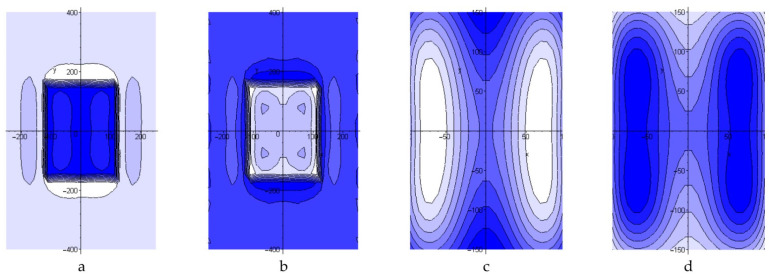
Distribution of normal stresses *σ_x_* in the aluminium panel (**a**,**b**) and repair patch mounted without preliminary dismantling (**c**,**d**) after completion of the temperature holding at *T_c_* and cooling, accordingly.

**Figure 9:**
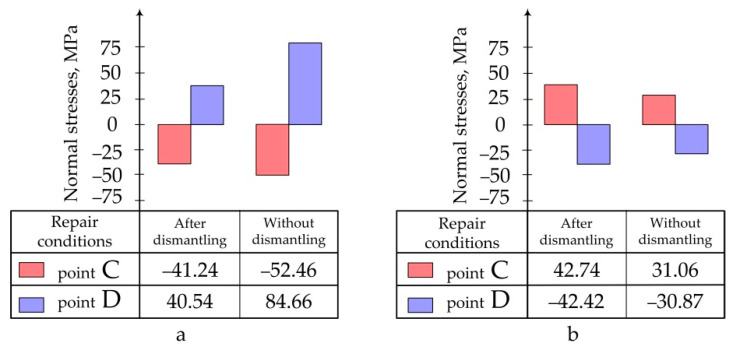
Maximum normal stresses, occurring in the aluminium panel (**a**) and composite patch (**b**) at various repair conditions.

For the mode No.5 (see Table 2) the stress–strain behaviour occurring in the process of repair of the structure under study without preliminary dismantling with the use of fine-fibre resistive structure in the heated tool was also calculated [19]. In this case, boundary conditions corresponded to the hinge mount of the structure along the contour. Setting of the temperature on the surface area being 15 mm larger than the patch on each side modelled the local heating using the thermal blanket [19]. Figure 8 shows the quality picture of the distribution of normal stresses in the aluminium panel under repair and patch at the stage of heating and after cooling of the structure. Figure 9 shows the maximum values of normal stresses arising in the structure under various repair conditions.

Based on the results of theoretical calculations, the conclusions below can be drawn. Curing in accordance with the standard mode (No. 1 in Table 2) recommended by the manufacturer of the binder EA9396 leads to an extremely low degree of curing and, consequently, strength of the repair patch. The need to ensure high physico-mechanical properties and stability of the PCM structures requires additional heat treatment.

Increase in the moulding temperature leads to the reduction in residual stresses in the structure being repaired. However, it significantly increases the current stresses at the stages of heating and temperature holding (Figure 6). Since physico-mechanical characteristics of the PCM patch at this stage are not high enough, there is a risk of the structure failure even in the curing process. Therefore, when choosing the curing temperature, it is important to take into account both the final and the current values of stresses in the structure under repair.

The binder heating rate increase leads to higher residual stresses in the structure under repair (Figure 7), since a slight expansion at the heating stage is insufficient to compensate for stresses arising in the process of cooling.

In theory, at certain combinations of the heating rate and curing temperature, zero residual stress structure can be obtained.

The conditions for repair works, such as fixation of the structure under repair, the heating method, etc., are to be taken into account when choosing the curing mode.

Shortening of the repair patch curing time and, consequently, reduction of energy consumption can be achieved through timely completion of the temperature holding.

In conclusion, it should be noted that the study and selection of the patch curing mode should be carried out for a specific structure, taking into account the nature of the repair process.

## 4. Experimental Research

To confirm the reliability of the results, as well as the conclusions made on their basis, stress–strain behaviour of the structure being repaired under several different curing modes has been studied experimentally.

**Figure 10:**
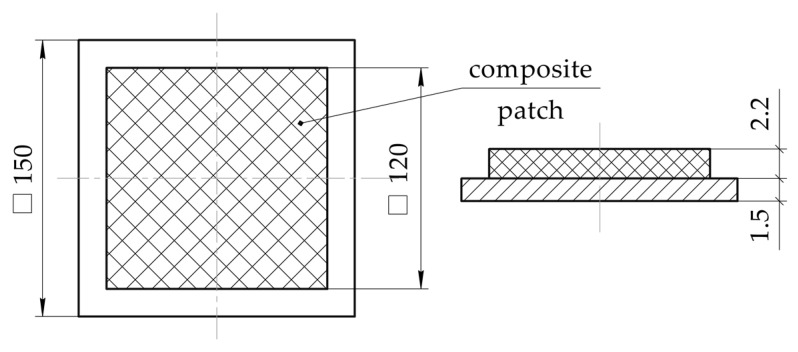
Geometric parameters of the experimental specimen.

To do this, carbon fibre composite patch (EA9396/Carbon 3K-70-P) with a lay-up pattern of [0°; 90°] was laid on a prefabricated specimen of the aluminium alloy 2024T3 by wet method. Thickness of the patch after curing was equal to 2.2 ± 0.07 mm. Geometric parameters of the test specimen are shown in Figure 10. Materials used in the experiment are similar to those used above (see Table 1).

For experimental curing of the repair patch, four modes were chosen (Table 3), previously investigated above (see Table 2).

**Figure 11:**
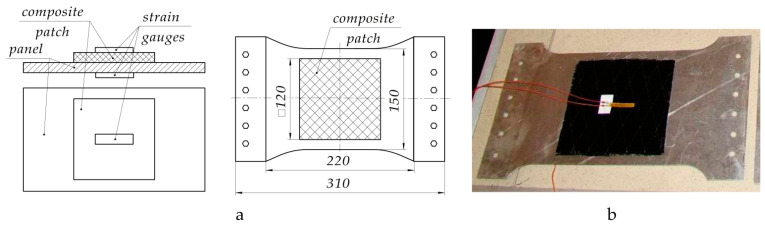
Diagram (**a**) and photo (**b**) of strain gauge mounted for the measurement of deformations in the process of patch curing.

Deformations occurring in the tested structure in the process of curing the composite patch were measured by KF5P1-20-400 strain gauges, mounted as shown in Figure 11.

Taking into account that the strain gauge allows obtaining the average value of the deformations only within its base [53]:

- Sensors were located directly in the centre of the aluminium plate and patch, where the deformation field is the most uniform one;
- During processing of analytical results, averaged deformations $\varepsilon^{\prime }$ were calculated as follows:
  (7) ε′=1l∫l2l2εx(x,y)|y=0dx,
  where l—strain gauge base length.

**Figure 12:**
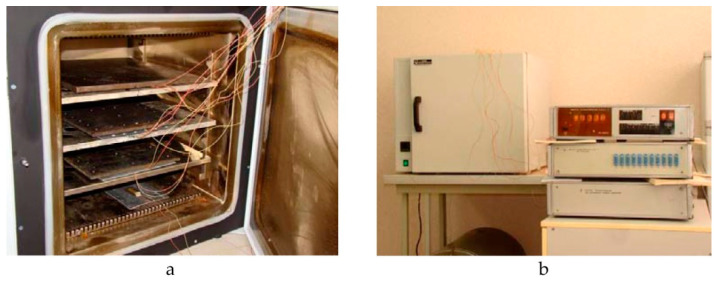
Research equipment: (**a**)—thermal oven; (**b**)—strain gauge measuring system of SIIT type.

To implement the selected curing modes of the repair patch, the thermal oven (Figure 12) providing the uniform heating over the entire area of the specimen was used. For more accurate temperature control, additional thermocouple was installed directly on the test specimen. Due to the small thickness of the specimen and two-way heating provided in the oven, uneven temperature distribution over the thickness was not taken into account.

To prevent the impact of internal temperature deformations of the strain gauge on the results of the experiment, strain gauges were connected to the SIIT measuring system taking into account the thermal compensation.

Boundary conditions in the calculations corresponded to the specimen fixation at the central point, which allowed its free deformations in all directions. As the factors contributing to occurrence of stresses, the following factors were considered:

- Distributed load of 0.0008 MPa equivalent to the action of 11.5 kg load on the patch;
- Temperature change in the curing process;
- Binder shrinkage.

**Figure 13:**
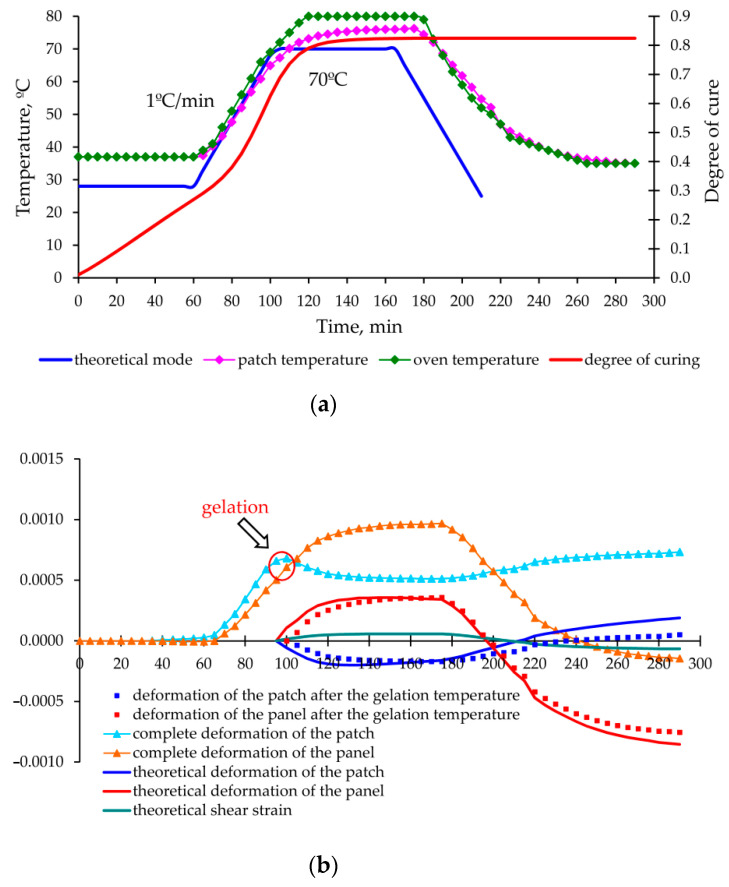
Change in the temperature, degree of curing (**a**) and deformations occurring (**b**) in the aluminium panel under repair and composite patch in the process of curing according to the mode 1 (Table 3).

**Figure 14:**
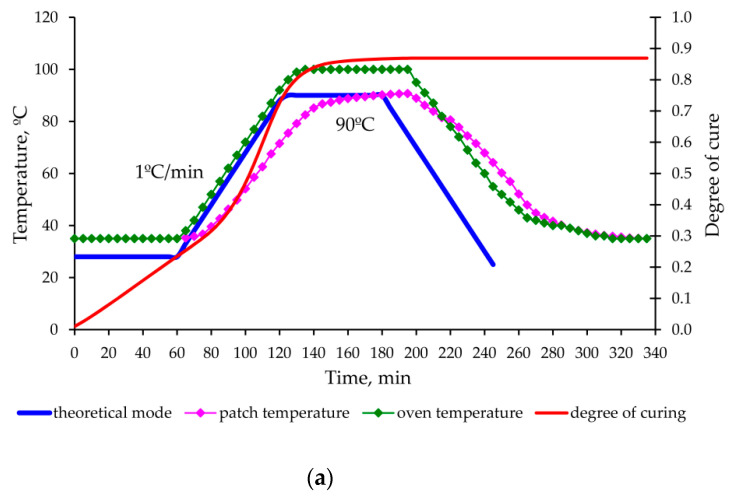

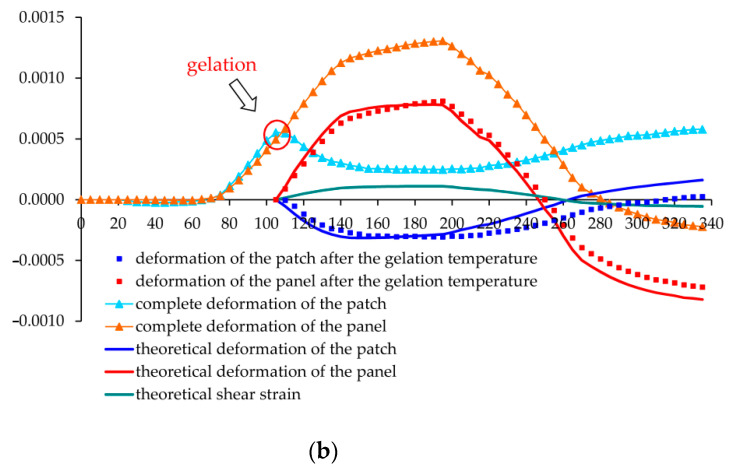
Change in the temperature, degree of curing (**a**) and deformations occurring (**b**) in the aluminium panel under repair and composite patch in the process of curing according to the mode 2 (Table 3).

**Figure 15:**
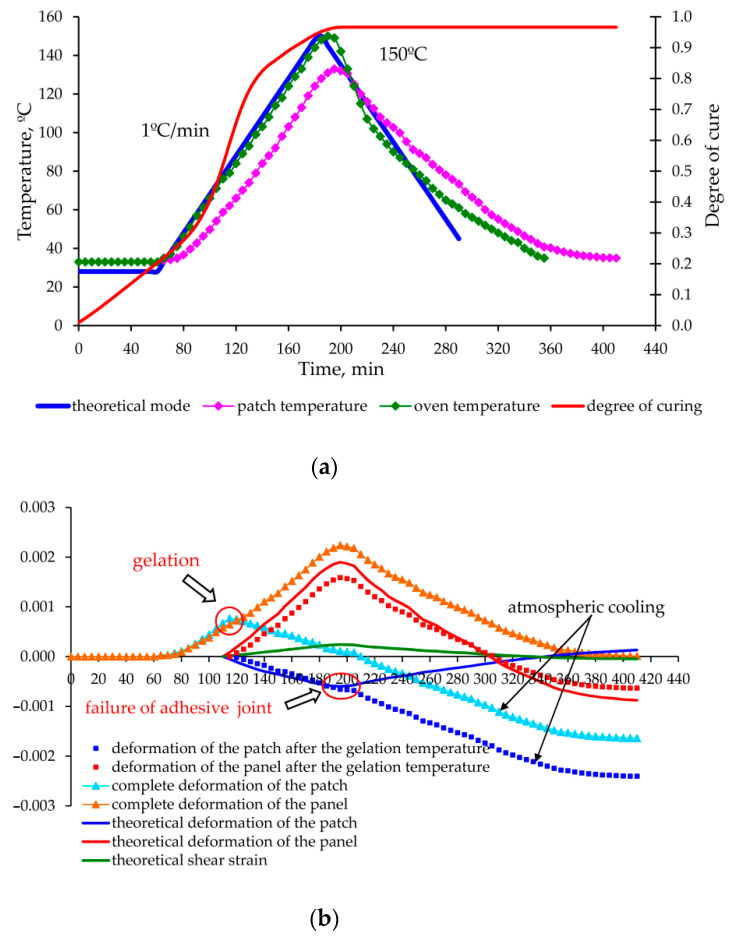
Change in the temperature, degree of curing (**a**) and deformations occurring (**b**) in the aluminium panel under repair and composite patch in the process of curing according to the mode 3 (Table 3).

**Figure 16:**
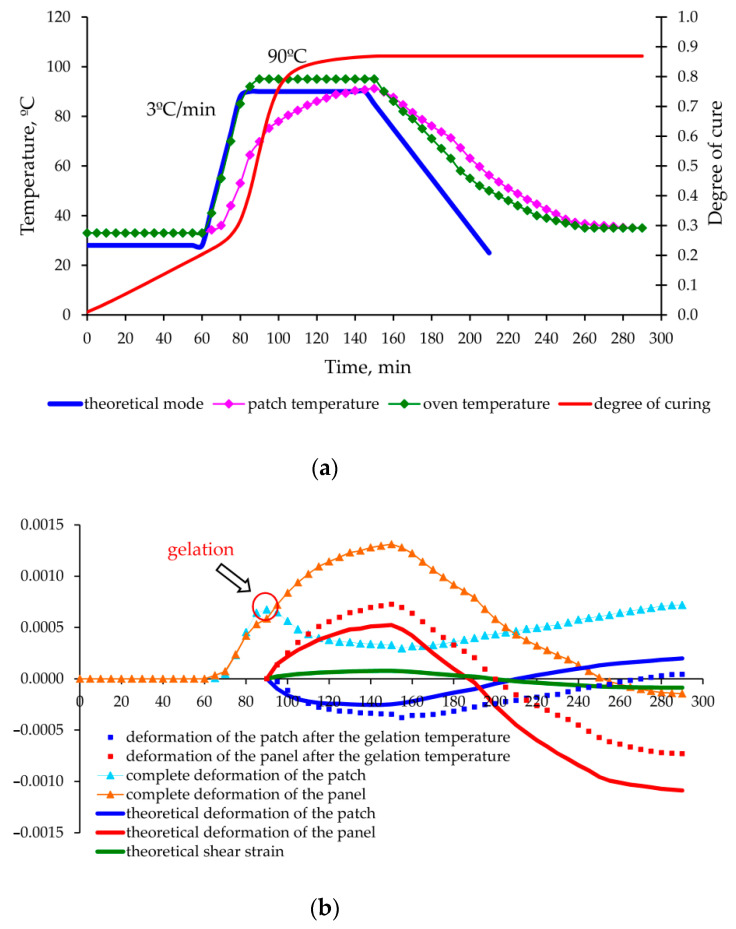
Change in the temperature, degree of curing (**a**) and deformations occurring (**b**) in the aluminium panel under repair and composite patch in the process of curing according to the mode 4 (Table 3).

As a result of experimental data processing and theoretical calculations made for each mode, graphs of deformation changes in the aluminium panel and carbon fibre composite patch were obtained during the entire moulding process, shown in Figure 13, Figure 14, Figure 15 and Figure 16.

Numerical values of deformations at control points C (holding completion of the cooling at $T=T_{c}$) and D (completion of cooling stage) for all investigated modes are summarized and analysed in Table 4.

After the analysis of the experimental results and their comparison with analytical solutions, we can state the following.

The proposed method for determination of the stress–strain behaviour of the structure in the moulding process, based on the analytical model of step-variable thickness [19] adequately describes the growth of deformations and stresses at the stage of heating and holding (experiments No. 1, 2 and 3—Figure 13, Figure 14 and Figure 15).

The assumptions used in modelling of the PCM properties, which are changing in the curing process, represent the cause of larger errors in calculating the repair patch deformations as compared to the deformations of the aluminium plate with the constant physical and mechanical characteristics.

Significant discrepancies between the theoretical and experimental values obtained in experiment No. 4 (Figure 16) after completion of the heating/holding stage are explained by the inaccuracy of modelling of the shrinkage of the EA9396 cold cure binder at high rates of temperature rise.

Increase in the difference between the experimental and theoretical values at the cooling stage is explained by the presence of relaxation processes, which were not taken into account in the theoretical calculations because of lack of rheological characteristics of the materials used. Taking these processes into account will improve the calculation accuracy, as well as provide an additional opportunity to optimize the cooling mode (selection of temperature holdings) and to further reduce the level of residual stresses.

The permissible level of residual stresses in the repaired structure is not a guarantee of high-quality repair, since the current stresses can significantly exceed the residual stresses and lead to appearance of hidden defects (cracks, delamination) or even the failure of the repair joint already in the curing process (as in experiment No. 2 Figure 14).

## 5. Discussion

Therefore, the new method for determination of technological stresses occurring in the structure under repair in the process of the composite patch curing has been developed. This method is based on the analytical model previously developed in [47] to determine the stress–strain behaviour of the panel of step-variable thickness. The research demonstrated the significant influence of the temperature–time regime of curing and methods of its implementation on the bearing capacity of the structure under repair. Results of the research allowed for the considerable improvement of the model of thermo-reactive binder curing obtained earlier in [23,25,26]. Consequently, it is now possible to obtain a more complete picture of the changes concerned and to determine the current and residual stress–strain behaviour of the structure under repair.

Our studies, which confirm the findings of [24,25,26,27], prove that at, the time of gelation, the degree of binder curing is a constant characteristic of the polymer not depending on the curing mode. For the epoxy binder Hysol EA9396 (Henkel) [48] under study, the degree of its curing is 52%.

When analysing the results, it is necessary to note the significate rise in stresses and more uneven stress distribution in the process of repair of the panel without preliminary dismantling, as proposed in [19]. Therefore, owing to the high requirements for the bearing capacity of the structure being repaired, the performance of repairs that work in this way requires more careful choice of the curing mode parameters; in extreme cases, it may not be acceptable at all.

The obtained experimental graph of total deformation of the composite patch allowed the researchers to clearly determine the moment of stress occurrence in the structure under repair. This moment matches quite exactly (with the discrepancy not exceeding 5 min) the gel point determined analytically based on dependence of the degree of curing on the moulding mode. It confirms the validity of the hypothesis accepted based on [26,27,29,49] on the occurrence of technological stresses in the structure after gelation only.

The developed method allows for increasing the repair efficiency [7,12,47] by taking into account the temperature–time regime of curing and methods of its implementation. The advanced comprehensive algorithm for choosing the effective parameters of the damaged panel repair process can be represented as follows.

1. In order to ensure the minimum downtime of the structure, and taking into account high complexity of dismantling operations, at the first stage the repair without preliminary dismantling is chosen.
2. Taking into account the limitations related to the quality of surface being repaired, the method of the repair patch installation is chosen.
3. The choice of materials to be used for repairs should, first of all, be guided by their physico-mechanical characteristics, availability and abundance. Much attention is also paid to the optimal combination of the mechanical and strength characteristics of the structure being repaired, the patch and adhesive used.
4. With the use of the current methods, geometric parameters of the repair patch, which ensure the restoration of initial bearing capacity of the structure, are determined.
5. For the resulting repair patch geometry, parameters of the curing mode giving rise to occurrence of the minimum residual technological stresses are defined with the use of the developed model. At this time, control of the current stresses in the structure under repair to prevent the failure of adhesive joint at the stage of repair is mandatory.
6. The impact of residual technological stresses on the bearing capacity of the repaired structure under the action of operational loads is assessed. If the total value of operational and technological stresses exceeds the permissible level, the following steps to adjust the repair patch geometric parameters are taken:
  - If the presence of the residual technological stresses leads to the failure of adhesive layer, plan dimensions of the patch should be increased and/or bevelling of edges is used to reduce the operational shear stresses;
  - When the bearing capacity of the structure under repair decreases in general, thickness of the repair patch should be increased. It contributes to the redistribution of stresses and the reduction of the load perceived by the panel. In this case, it can also be necessary to increase plan dimensions of the patch to satisfy the conditions of adhesive layer strength;
  - Similar actions are to be taken in case of the violation of the conditions of repair patch strength as well.
  - As evidenced in practice, there is no need to repeatedly optimize the curing mode in case of slight adjustment of the repair patch dimensions. Therefore, residual technological stresses occurring in the structure with the adjusted geometry during moulding are determined in accordance with parameters of p. 5, and their acceptability is assessed.
  - The sequence of actions described in p. 4–7 should be followed until the set of parameters of the repair process is determined (repair patch geometry + curing mode), which allows for satisfying a condition of restoration of the structure bearing capacity. If no such parameters are found during the search, it is necessary to:
  - Return to p. 3 and choose the materials (adhesive and/or PCM) with the higher strength characteristics, and then continue to search for parameters according to the described algorithm;
  - When the other materials are not available, or the condition of restoration of the bearing capacity still cannot be satisfied with the use of such materials, it is possible to choose the repair of dismantled panel, i.e., to return to p. 1. However, it is important to note that it is the least effective method in terms of labour costs and time required;
  - If in the course of repair of the dismantled panel the bearing capacity fails to reach the specified value, the repair is considered inexpedient, and the damaged structure is replaced by a new one.

Thus, the research, together with the results previously obtained [19,47], allows researchers to make an integrated choice of geometric parameters of the repair composite patch and temperature–time regime of its curing in order to ensure the specified level of strength and stiffness of the structure under repair.

## 6. Conclusions and Further Research

The method for determination of technological stresses, which arise in the structure under repair in the process of curing of the composite patch, has been developed. This method allows one to:

- Take into account the shrinkage, change in physico-mechanical characteristics and rheological processes occurring in the binder during the moulding process;
- Determine the stresses in the structure being repaired at any time, which allows avoiding premature failure of the adhesive joint at the repair stage;
- Take into consideration the impact of the conditions of repair works.

Reliability of assumptions and developed method is confirmed by comparison with the experimental data.

Our studies demonstrated a significant effect of the temperature–time regime of curing and the methods of its implementation on the bearing capacity of the structure being repaired. Nevertheless, in order to increase the economic efficiency of the operation of the repaired structures of various applications, it is necessary when choosing these parameters to additionally take into account the factors of the cost of materials and repair works, increase in operating expenses as a result of higher mass of the structure and downtime.

## Figures and Tables

**Table 1:** Physico-mechanical characteristics of initial PCM components.

	Initial Components	Binder EA9396	Carbon Fibre
Characteristics			
Elastic modulus, MPa		2750	290,000
Poisson’s ratio		0.35	0.07
Linear thermal expansion coefficient *α*·10^−6^, 1/C		85 at *T* ≤ 65 °C 75 at 65 °C < *T* ≤ 95 °C 70 at 95 °C < *T* ≤ 105 °C 65 at 105 °C < *T* ≤ 125 °C 50 at *T* > 125 °C	−0.64
Volumetric content in PCM		0.45	0.55

**Table 2:** Parameters of analysed curing modes.

Number of Regime	Heating Rate *V*, °C/min	Curing Temperature *T_c_*, °C	Curing Time *t_c_*, °min	Note
1	–	25	7200	Standard mode recommended by the manufacturer [49]
2	1	90	60	Mode No.1 + additional heat treatment
3	1	66	60	Accelerated mode, recommended by the manufacturer
4	1	70	60	–
5	1	90	60	–
6	1	120	60	–
7	1	150	–	–
8	0.5	90	60	–
9	3	90	60	–
10	5	90	60	–

**Table 3:** Parameters of the repair patch curing modes.

Number	Curing Mode
1	Holding at *T* = 28 °C (1 h); heating to *T* = 70 °C at the rate of 1 °C/min;
	holding at *T* = 70 °C (1 h); atmospheric cooling
2	Holding at *T* = 28 °C (1 h); heating to *T* = 90 °C at the rate of 1 °C/min;
	holding at *T* = 90 °C (1 h); atmospheric cooling
3	Holding at *T* = 28 °C (1 h); heating to *T* = 150 °C at the rate of 1 °C/min;
	atmospheric cooling
4	Holding at *T* = 28 °C (1 h); heating to *T* = 90 °C at the rate of 3 °C/min;
	holding at *T* = 90 °C (1 h); atmospheric cooling

**Table 4:** Comparison of results of theoretical calculations and experimental studies.

Point	Deformations in Carbon Fibre Composite Patch			Deformations in Aluminium Panel			Shear Deformation
	Exp.	Analytical	Error, %	Exp.	Analytical	Error, %	Analytical
Curing mode 1							
C	−1.69 × 10^−4^	−1.60 × 10^−4^	5.79%	3.59 × 10^−4^	3.43 × 10^−4^	4.70%	5.31 × 10^−5^
D	5.05 × 10^−5^	1.89 × 10^−4^	—	−7.55 × 10^−4^	−8.53 × 10^−4^	12.29%	−6.21 × 10^−5^
Curing mode 2							
C	−3.06 × 10^−4^	−2.85 × 10^−4^	7.07%	8.11 × 10^−4^	7.78 × 10^−4^	4.12%	1.10 × 10^−4^
D	2.63 × 10^−5^	1.61 × 10^−4^	—	−7.19 × 10^−4^	−8.21 × 10^−4^	13.35%	−5.47 × 10^−5^
Curing mode 3–Failure of adhesive joint							
C	−6.56 × 10^−4^	−6.01 × 10^−4^	8.82%	1.59 × 10^−3^	1.90 × 10^−3^	17.78%	2.40 × 10^−4^
D	−2.41 × 10^−3^	1.31 × 10^−4^	—	−6.35 × 10^−4^	−8.79 × 10^−4^	—	−4.57 × 10^−5^
Curing mode 4							
C	−3.46 × 10^−4^	−2.49 × 10^−4^	32.48%	7.27 × 10^−4^	5.24 × 10^−4^	32.35%	7.92 × 10^−5^
D	4.41 × 10^−5^	1.99 × 10^−4^	—	−7.30 × 10^−4^	−1.09 × 10^−3^	39.43%	−8.71 × 10^−5^

## Data Availability

Not applicable.
